# Characterization of a Novel Thermostable Dye-Linked l-Lactate Dehydrogenase Complex and Its Application in Electrochemical Detection

**DOI:** 10.3390/ijms222413570

**Published:** 2021-12-17

**Authors:** Takenori Satomura, Kohei Uno, Norio Kurosawa, Haruhiko Sakuraba, Toshihisa Ohshima, Shin-ichiro Suye

**Affiliations:** 1 Division of Engineering, Faculty of Engineering, University of Fukui, Fukui 910-8507, Japan; suyeb10@u-fukui.ac.jp; 2 Life Science Innovation Center, University of Fukui, Fukui 910-8507, Japan; 3 Department of Applied Chemistry Biotechnology, Graduate School of Engineering, University of Fukui, Fukui 910-8507, Japan; unok@biofiber-fukui.com; 4 Department of Science and Engineering for Sustainable Innovation, Faculty of Science and Engineering, Soka University, Tokyo 192-8577, Japan; kurosawa@soka.ac.jp; 5 Department of Applied Biological Science, Faculty of Agriculture, Kagawa University, Takamatsu 761-0795, Japan; sakuraba.haruhiko@kagawa-u.ac.jp; 6 Department of Biomedical Engineering, Faculty of Engineering, Osaka Institute of Technology, Osaka 535-8585, Japan; toshihisa.oshima@oit.ac.jp

**Keywords:** dye-linked l-lactate dehydrogenase, FMN, hyperthermophilic archaeon, thermostable enzyme, heterogeneous expression

## Abstract

Flavoenzyme dye-linked l-lactate dehydrogenase (Dye-LDH) is primarily involved in energy generation through electron transfer and exhibits potential utility in electrochemical devices. In this study, a gene encoding a Dye-LDH homolog was identified in a hyperthermophilic archaeon, *Sulfurisphaera tokodaii*. This gene was part of an operon that consisted of four genes that were tandemly arranged in the *Sf. tokodaii* genome in the following order: *stk_16540*, *stk_16550* (dye-ldh homolog), *stk_16560,* and *stk_16570*. This gene cluster was expressed in an archaeal host, *Sulfolobus acidocaldarius*, and the produced enzyme was purified to homogeneity and characterized. The purified recombinant enzyme exhibited Dye-LDH activity and consisted of two different subunits (products of stk_16540 (α) and stk_16550 (β)), forming a heterohexameric structure (α3β3) with a molecular mass of approximately 253 kDa. Dye-LDH also exhibited excellent stability, retaining full activity upon incubation at 70 °C for 10 min and up to 80% activity after 30 min at 50 °C and pH 6.5–8.0. A quasi-direct electron transfer (DET)-type Dye-LDH was successfully developed by modification of the recombinant enzyme with an artificial redox mediator, phenazine ethosulfate, through amine groups on the enzyme’s surface. This study is the first report describing the development of a quasi-DET-type enzyme by using thermostable Dye-LDH.

## 1. Introduction

Dye-linked l-lactate dehydrogenase (Dye-LDH) is a flavoenzyme containing FMN as a cofactor, which catalyzes oxidation of l-lactate to pyruvate in the presence of natural electron acceptors such as cytochrome *c*. Dye-LDH is widely distributed in bacteria and eukaryotes [1,2] and plays an important role in energy generation within cells as the primary enzyme in electron transfer systems. In addition, Dye-LDH can utilize artificial electron acceptors, such as 2,6-dichlorophenolindophenol (DCIP) and potassium ferricyanide, in place of its natural electron acceptor. These artificial electron acceptors can efficiently transfer electrons derived from substrates to electrodes. Therefore, Dye-LDH exhibits significant potential for utilization as a specific element in electrochemical devices, such as l-lactate biosensors and l-lactate biofuel cells [3,4,5,6,7,8,9]. Despite its significant potential utility, practical applications of Dye-LDH have been limited as a result of its poor stability [8].

Hyperthermophiles, which can grow at or near the boiling point of water, are excellent sources of microbial enzymes that are highly stable under a variety of conditions, such as high temperature, acidity, and alkalinity [10,11,12]. It is, therefore, worth exploring hyperthermophiles as potential sources of stable Dye-LDH for electrochemical applications.

To this end, we explored available genomic information on hyperthermophiles and found a Dye-LDH homolog in the hyperthermophilic archaeon *Sulfurisphaera tokodaii*. The gene encoding the Dye-LDH homolog formed a gene cluster with other genes of unknown function in *Sf. tokodaii*. The gene cluster containing the Dye-LDH homolog gene was expressed in an archaeal host, *S. acidocaldarius* SK1. The produced protein exhibited Dye-LDH activity and formed a heteromeric structure with an unknown gene product within the Dye-LDH gene cluster. In this study, we performed detailed biochemical characterizations of this enzyme and examined its utility as a specific element for l-lactate electrochemical devices.

## 2. Results

### 2.1. Expression of Dye-LDH in S. acidocaldarius SK1

**Figure 1:**
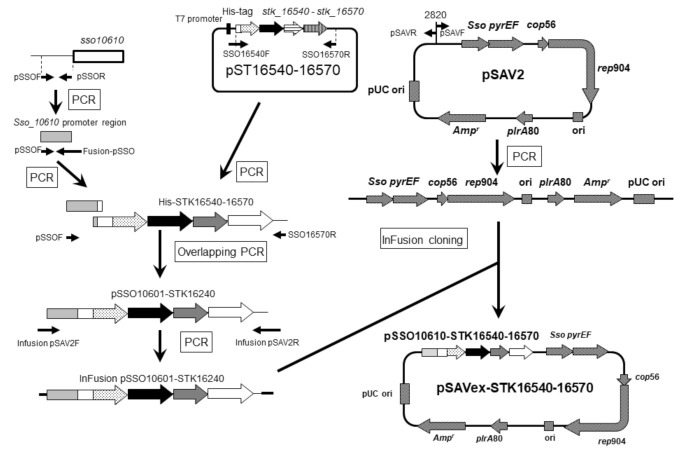
Strategy for construction of pSAVex-STK16540-16570.

*S. acidocaldarius* was used as a host for co-expression of *stk_16540*, *stk_16550*, *stk_16560,* and *stk_16570* genes from *Sf. tokodaii*. For this purpose, an expression plasmid pSAVex-STK16540-16570 was constructed on a pSAV2 vector backbone, with genes cloned from an *sso 10610* gene promoter (Figure 1).

The constitutive promoter *sso 10610* was selected for expression of the gene cluster in *S. acidocaldarius* SK1 cells based on a previous report [13]. *S. acidocaldarius* SK1-carrying pSAVex-STK16540-16570 was cultivated in an XT medium, and a crude extract prepared from these cells exhibited Dye-LDH activity, indicating successful production of the enzyme. The specific activity of the crude extract from *S. acidocaldarius* SK1 transformants was estimated to be 0.0156 units/mg (Table 1). Total protein of 0.441 mg of pure recombinant Dye-LDH was obtained from the crude extract of *S. acidocaldarius* SK1 cells in four liters of medium (Table 1).

**Figure 2:**
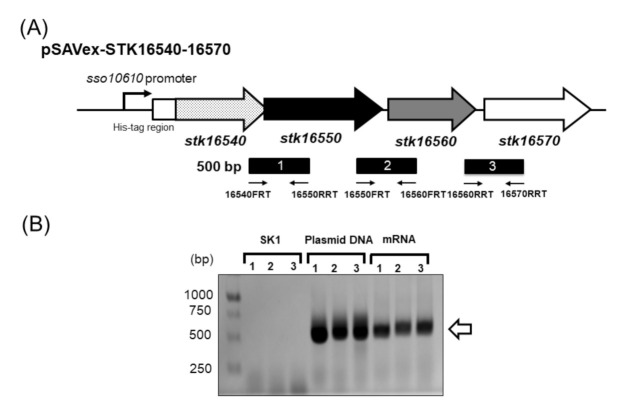
Strategy for construction of pSAVex-STK16540-16570. (**A**) Location of amplified regions obtained during RT-PCR experiments. Genes are indicated by arrows. Dotted, black, gray, and white arrows indicate *stk16540*, *stk16550*, *stk16560,* and *stk16570* genes, respectively. The line below the arrows indicates regions amplified in the RT-PCR experiments. (**B**) PCR products obtained from RT-PCR experiments. Genomic DNA from *S. acidocaldarius* SK1, pSAVex-STK16540-16570 plasmid DNA, and mRNA derived from *S. acidocaldarius*-carrying pSAVex-STK16540-16570 plasmid were used as templates. Numbers on the Y-axis indicate lengths (in bp) of protein standards. Expected amplicon length was 500 bp.

In order to confirm co-transcription of the gene cluster containing *stk_16540*, *stk_16550*, *stk_16560,* and *stk_16570* genes, RT-PCR was performed by using total RNA isolated from *S. acidocaldarius* SK1-carrying pSAVex-STK16540-16570 grown in XT medium. RT-PCR primers were designed to amplify intergenic regions between the four genes (Figure 2, Table 2). As a result, an amplification of the three intergenic regions was achieved (Figure 2), indicating that *stk_16540*, *stk_16550*, *stk_16560,* and *stk_16570* genes are located within the same transcription unit.

### 2.2. Purification and Molecular Composition of Recombinant Dye-LDH from S. acidocaldarius SK1 Carrying pSAVex-STK16540-16570

**Figure 3:**
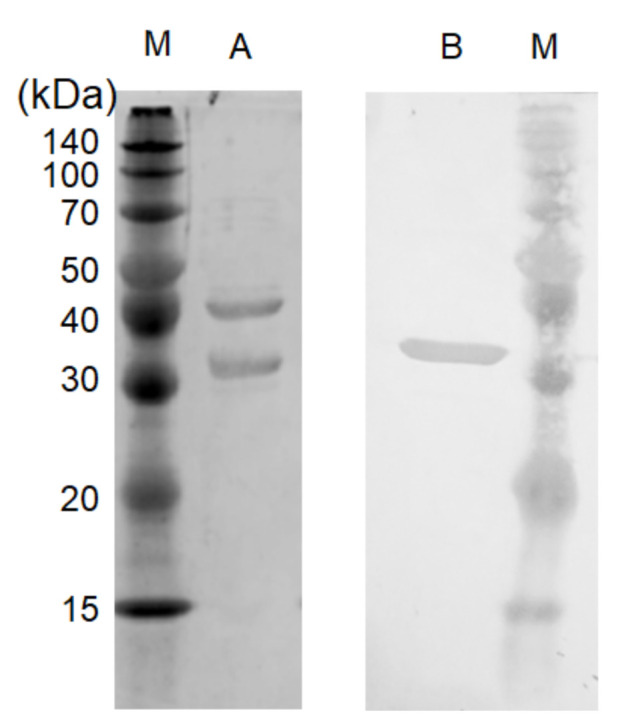
SDS-PAGE and Western blotting analysis of recombinant Dye-LDH. Lane M, markers; (**A**), Coomassie-stained gel of purified Dye-LDH; (**B**), Western blot of purified Dye-LDH developed by using Anti-His-tag mAb-HPR-DiercT.

The purification of recombinant Dye-LDH from *S. acidocaldarius* SK1-carrying pSAVex-STK16540-16570 is summarized in Table 1. The enzyme was purified from crude cell lysate in a two-step process involving HiTrap FF crude nickel chelating and HiTrap Q strong anion exchange chromatography. Dye-LDH was purified approximately 98.1-fold after the final step, with an overall yield of approximately 9.67%. The purified enzyme was homogenous, separating as a single protein based on native gradient PAGE. The native gradient PAGE system can estimate molecular masses of proteins based on a blue-native polyacrylamide electrophoresis technique [14,15,16]. Purified Dye-LDH was estimated to be approximately 253 kDa (Supplementary Figure S1) by the native gradient PAGE system. SDS-PAGE revealed two different bands, indicating that this enzyme consists of heterogeneous subunits (Figure 3). Molecular masses of small and large subunits were estimated to be approximately 30 kDa and 40 kDa, respectively. STK16540 protein was expressed as the fusion protein with *N*-terminal His-tag in the *S. acidocaldarius* recombinant protein expression system. Immunodetection with His-tag specific antibody indicated that the small subunit (30 kDa) was STK16540 (Figure 3). In addition, the molecular mass of the large subunit (40 kDa) coincided with that of the Dye-LDH homolog (43 kDa) encoded by the *stk_16550* gene. Together, these results indicate that the enzyme forms an α3β3 heterohexameric structure.

### 2.3. Detection of Prosthetic Group

In general, dye-linked dehydrogenases contain FMN or FAD as cofactors. A flavin compound was extracted from the purified Dye-LDH complex in this study and analyzed by using high-performance liquid chromatography. Consequently, the flavin compound in the enzyme extract was identified as FMN (Supplementary Figure S2).

**Figure 4:**
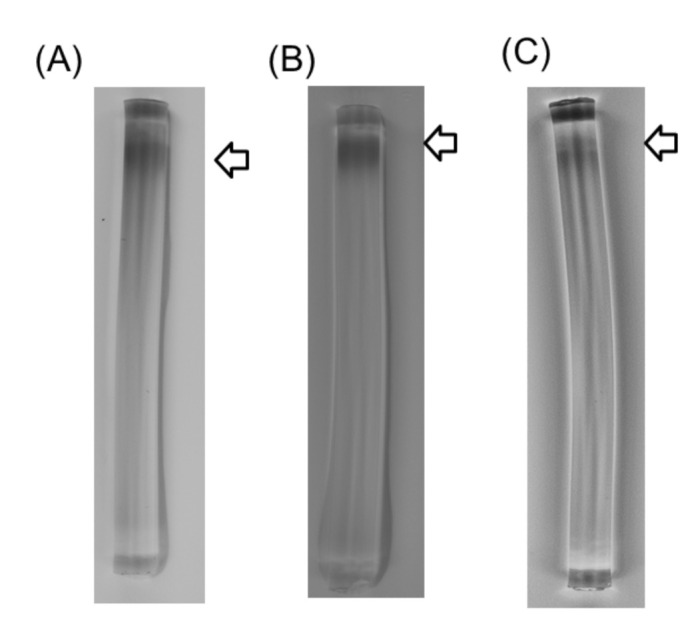
Native PAGE of purified Dye-LDH. Coomassie staining (**A**), activity staining (**B**), and non-heme iron staining (**C**). Arrows indicate locations of Dye-LDH bands.

In addition, the presence of iron-sulfur cluster motifs was predicted from information on the Dye-LDH’s primary structure. In order to detect iron in Dye-LDH, we performed specific staining for non-heme iron on polyacrylamide gels. A specific stained band was detected in the gel, with mobility consistent with corresponding Dye-LDH bands identified by Coomassie staining and Dye-LDH activity staining (Figure 4).

### 2.4. Effect of pH and Temperature on Dye-LDH Activity and Stability

Recombinant Dye-LDH exhibited maximum activity at pH 6.5. Enzyme activity also increased linearly with increasing temperatures from 40 °C to 80 °C. Unfortunately, further increases in temperature resulted in non-enzymatic decolorization of DCIP, which interfered with the assay. The enzyme retained over 85% of its original activity after incubation for 10 min at temperatures below 70 °C and over 45% of its activity even after incubation for 10 min at 90 °C. When the enzyme was incubated for 30 min at 50 °C at different pH ranging from 6.5 to 8.0, more than 80% of its initial activity was retained.

### 2.5. Electron Donor and Acceptor Specificities

In addition to l-lactate, several other hydroxy acids served as electron donors. Dye-LDH activities observed with 2-hydroxyhexanoate, dl-2-hydroxybutyrate, l-mandelate, and l-3-phenyllactate were 130%, 116%, 22%, and 2.9%, respectively, when compared to activities observed with l-lactate. d-lactate was not used as an electron donor. Enzyme electron acceptor specificity was also examined. DCIP was the electron acceptor preferred by the enzyme for l-lactate oxidation. MTT, ferricyanide, a phenazine methosulfate/*p*-iodonitrotetrazolium violet-coupled system (PMS-INT), and a 1-methoxy phenazine ethosulfate/*p*-iodonitrotetrazolium violet-coupled system (PES-INT) were also used as electron acceptors, with relative activities of 11%, 6.2%, 0.79%, and 0.58%, respectively, when compared to activities observed with DCIP. O_2_, benzyl viologen, and methylene blue were inert as electron acceptors. Kinetic analysis yielded Km values of 0.199 mM for l-lactate as the electron donor and 0.2 mM for DCIP as the electron acceptor (Supplementary Figure S3)

### 2.6. Characterization of PES-Modified Dye-LDH Electrode

**Figure 5:**
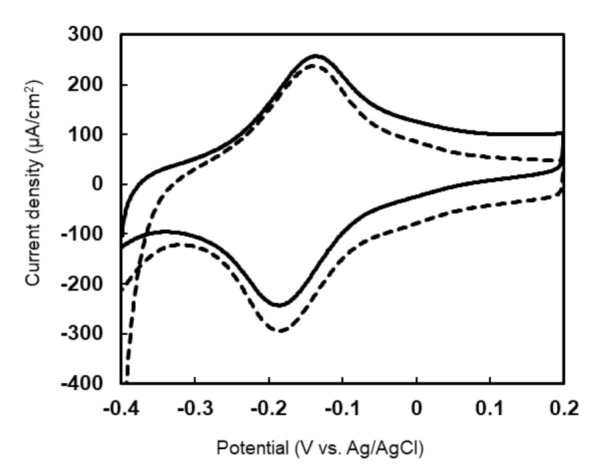
Cyclic voltammograms of PES-modified Dye−LDH electrode without (dashed line) and with (solid line) l-lactate in standard reaction solution at 50 °C.

An electrode with PES-modified Dye-LDH was prepared, and its characteristics were examined by using cyclic voltammetry (CV). Cyclic voltammograms of the PES-modified Dye-LDH electrode clearly showed oxidation and reduction peaks around −100 mV (vs. Ag/AgCl), indicating the presence of PES on the enzyme surface (Figure 5, dashed line). Furthermore, the PES oxidation peak increased in the presence of l-lactate (Figure 5, solid line). This result indicates that catalytic reactions through l-lactate oxidation produce electrons that react with the oxidized form of PES attached to Dye-LDH’s surface.

**Figure 6:**
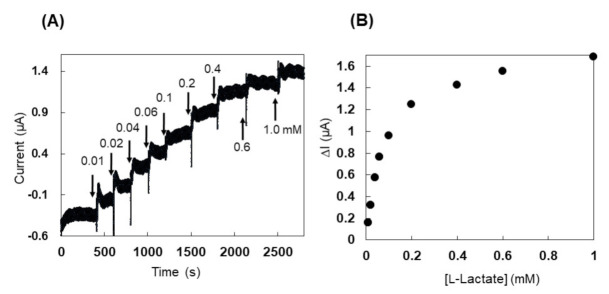
(**A**) Time course of chronoamperometry measurements with PES-modified Dye-LDH. Arrows indicate the addition of l-lactate, with total l-lactate concentration after addition displayed above them. (**B**) Dependence of response current on l-lactate concentration for PES-modified Dye-LDH. Application potential 0 mV vs. Ag/AgCl.

In addition, chronoamperometry (CA) measurements were conducted by using a PES-modified Dye-LDH electrode. CA measurements showed a clear response to the addition of l-lactate at an operation potential of 0 mV (vs. Ag/AgCl) (Figure 6A). Response currents increased linearly for l-lactate concentrations less than 0.06 mM and saturated at l-lactate concentrations greater than 0.6 mM (Figure 6B).

## 3. Discussion

In this study, we identified the gene encoding Dye-LDH from a thermophilic archaeon, *Sf. tokodaii*, and constructed a recombinant expression system for Dye-LDH by using thermoacidophilic archaeon *S. acidocaldarius* SK1 as a host. The recombinant protein was successfully expressed and purified and showed Dye-LDH activity. SDS-PAGE (Figure 3) and Western blotting analysis showed that purified Dye-LDH formed a heterohexameric α3β3 structure composed of a hypothetical protein (α STK16540) and a Dye-LDH homolog (β: STK16550).

Thus far, we have discovered two types of dye-lined l-proline dehydrogenase complexes in hyperthermophilic archaea: αβγδ heterotetrameric complexes and α4γ4 hetero-octameric complex [17,18,19,20]. These enzyme complexes were successfully expressed as recombinant proteins in *E. coli*, with promoter and Shine–Dalgarno sequences arranged upstream of the gene encoding each subunit. However, by using the *S. acidocaldarius* SK1 system in this study, genes encoding the Dye-LDH complex were easily expressed by using only a single promoter for the entire gene cluster. After expression in *S. acidocaldarius* SK1, the Dye-LDH complex was easily purified to homogeneity in two chromatography steps (Table 1, Figure 3). Although the Dye-LDH homolog gene has been found in genomes of bacteria and archaea, this study is the first, to our knowledge, to show the expression of the Dye-LDH complex as a recombinant protein and detailed characterization of its biochemical properties.

Currently, Dye-LDH, also known as flavocytochrome *b2*, has been isolated from yeast, and its biochemical properties and 3D structure have been investigated [2]. Yeast Dye-LDH containing FMN and heme *b2* as cofactors is a homotetramer and is localized in intermembrane spaces of mitochondria. Electron transfer mechanisms in yeast Dye-LDH have been revealed by using protein structural analysis. Electrons obtained through oxidation of l-lactate via enzyme reactions are first received by FMN, transferred to heme *b2,* and then accepted by cytochrome *c* via heme *b2* in the enzyme. Eventually, reduced cytochrome *c* generates energy through electron transfer systems in mitochondria. In this study, we revealed that the *Sf. tokodaii* Dye-LDH complex contained FMN and Fe (Figure 4). In addition, the primary structure of STK 16540 and STK16550 proteins composing the Dye-LDH complex contained the Fe-S cluster motif. These results suggest that Dye-LDH utilizes FMN and Fe-S clusters as cofactors, which are likely associated with electron transfer systems.

With regard to its structure and electron transfer mechanism, archaeal Dye-LDH may be inherently different from eukaryotic Dye-LDH reported previously. Detailed comparisons of the structure and function of both Dye-LDHs may provide further information about a potentially novel electron transfer mechanism via l-lactate oxidation.

We further studied the applicability of the Dye-LDH enzyme in mediating electron transfer. This was tested by fabricating a PES-modified Dye-LDH, which was modified with a redox mediator, amine-reactive PES (arPES). arPES is functionalized with a succinimide group and spontaneously binds to amine groups on the enzyme’s surface, thereby enabling the introduction of a quasi-DET ability to Dye-LDH (Supplementary Figure S4). Generally, a suitable free redox mediator is required for electrochemical detection by using dye-linked dehydrogenases. However, chronoamperometric analysis by using PES-modified Dye-LDH can detect l-lactate without the addition of any free redox mediator in the measurement system (Figure 6).

Currently, electrochemical detection through a quasi-DET reaction by using PES-modified dye-linked dehydrogenases has been reported for glucose dehydrogenase and l-lactate dehydrogenase [21,22,23,24]. To our knowledge, this is the first example of a quasi-DET response of a PES-modified thermostable Dye-LDH. Enzymes from thermophiles are known to be significantly more stable at room temperature than their counterparts from mesophiles [10,11,12]. Therefore, the redox mediator-modified thermostable Dye-LDH exhibits significant potential for application as a long-term measurable l-lactate biosensor. Further studies on practical applications of PES-modified Dye-LDH in l-lactate biosensors are currently underway.

## 4. Materials and Methods

### 4.1. Materials

Lithium l-lactate was obtained from Sigma-Aldrich (St. Louis, MO, USA). DCIP was purchased from FUJIFILM Wako Pure Chemical Corporation (Osaka, Japan). Tryptone and yeast extract were obtained from Difco Laboratories (Sparks, MD, USA). arPES was purchased from Dojjindo Laboratories (Kumamoto, Japan). Multi-walled carbon nanotubes (MWCNTs, 10–20 nm in diameter and 5–15 µm in length) were obtained from Tokyo Chemical Industry (Tokyo, Japan). All other chemicals were of reagent grade.

### 4.2. Strains and Cultivation Media

*S. acidocaldarius* SK1-carrying Δ*pyrEF* and Δ*sauI* [25] was used for production of the recombinant protein and cultivated on xylose and tryptone (XT) medium [26]. Solid XT medium was prepared by adding 0.65% (*w*/*v*) Gelrite (FUJIFILM Wako, Osaka, Japan), 1 mM CaCl_2_·2H_2_O, and 2 mM MgSO_4_·7H_2_O. *E. coli* strain DH5α (Agilent Technologies, Santa Clara, CA, USA) was used for plasmid cloning.

### 4.3. Plasmid Construction for Gene Expression in S. acidocaldarius SK1

In order to clone the Dye-LDH homolog gene cluster (*stk_16540*, *stk_16550*, *stk_16560*, and *stk_16570*), we first constructed a pST16540-16570 plasmid (Figure 1) by using a pET15b vector backbone (Novagen, Madison, WI, USA). A 4-kilobase gene fragment containing the Dye-LDH homolog gene was amplified from *Sf. tokodaii* genomic DNA with primers pET16540F and pET16570R (Table 2). The resultant PCR product was designated Infusion-STK16540-16570. The pET15b plasmid was linearized by PCR with primers pET15bF and pET15bR (Table 2) by using PrimeSTAR Max DNA polymerase (Takara Bio, Shiga, Japan). The intact pET15b template was removed by *DpnI* digestion. Infusion-STK16540-16570, containing a 15-base pair overlap sequence corresponding to the ends of the linearized pET15b plasmid, was inserted into the linearized pET15b vector by using an In-Fusion HD cloning kit (Takara Bio, Shiga, Japan) to generate pST16540-16570 according to the manufacturer’s protocol.

The strategy for constructing the Dye-LDH homolog gene cluster expression plasmid for *S. acidocaldarius* SK1 is shown in Figure 1. The gene cluster was amplified from pST16540-16570 with primers SSO16540F and SSO16570R (Table 2) by using PrimeSTAR Max DNA polymerase to generate DNA fragment His-STK16540-16570 (Figure 1).

The sso10610 gene promoter region (-219 to -1) was amplified by using the primer pair pSSOF and pSSOR (Table 2) from the genomic DNA of *Saccharolobus solfataricus* as a template. The resultant PCR product was used as the template in the next PCR by using the primer pair pSSOF and Fusion-pSSO (Table 2).

The primers Fusion-pSSO and SSO16570F were designed to contain overhanging ends of the *sso10610* promoter region and His-STK16540-16570 (Figure 1). The *sso 10610* promoter region and His-STK16540-16570 were fused by overlapping PCR by using the primer pair pSSOF and SSO16570R to generate the fragment pSSO10601-STK16240 [27]. A DNA fragment, InFusion-pSSO10601-STK16240, containing a 15-base pair overlap sequence corresponding to the ends of the linearized pSAV2 plasmid was amplified from pSSO10601-STK16240 with primers Infusion pSAV2F and Infusion pSAV2R (Table 2). InFusion-pSSO10601-STK16240 was then inserted into the linearized pSAV2 treated with *Dpn*I by using an In-Fusion HD cloning kit (Takara Bio, Shiga, Japan) to generate pSAVex16540-16570 according to the manufacturer’s protocol.

### 4.4. Total RNA Preparation and Reverse Transcription PCR

Total RNA was isolated from *S. acidocaldarius* SK1 harboring pSAVex16540-16570 cultivated in XT medium by using RNeasy Plus Universal Kits (Qiagen, Venlo, Netherlands). Reverse transcription PCR (RT-PCR) was performed by using the RT-PCR Quick Master Mix (Toyobo, Osaka, Japan). RT-PCR primers were designed to amplify intergenic regions *stk_16540*-*stk_16550*, *stk_16550*-*stk_16560*, and *stk_16560*-*stk_16570* (Table 2, Figure 1). Reactions were assembled according to the manufacturer’s protocol by using 490 ng total RNA in a 50-microliter reaction mixture. RNA concentration was adjusted by using NanoVue Plus (GE Healthcare, Buckinghamshire, UK).

### 4.5. Determination of Dye-LDH Activity and Protein Concentrations

Dye-LDH activity was determined by using a spectrophotometric assay. The reaction mixture contained 40 mM l-lactate lithium salt, 200 mM PIPES-NaOH buffer (pH 6.5), 0.2 mM DCIP, and an appropriate amount of enzyme in a total volume of 1 mL. The mixture without DCIP was incubated at 60 °C for 3 min in a cuvette with a 4-mm light path, and the reaction was started by adding DCIP, after which the initial decrease in absorbance at 600 nm was measured. One unit of enzyme was defined as the amount catalyzing the reduction of 1 µmol DCIP per min at 60 °C. A millimolar absorption coefficient of 19.1 mM^−1^ cm^−1^ at 600 nm was used for DCIP. Protein concentration was determined by using a Pierce™ Coomassie (Bradford) Protein Assay Kit (Thermo Scientific, Rockford, IL, USA), with bovine serum albumin serving as the protein standard.

### 4.6. Expression and Purification of Recombinant Dye-LDH from S. solfataricus

*S. acidocaldarius* SK1 was transformed with the expression plasmid pSAVex16540-16570 by electroporation according to a previously described method [23]. Transformed cells were plated onto a solid XT medium and cultivated at 70 °C for 10 days. Transformants were pre-cultured at 70 °C for 4 days in 10 mL of XT medium and then transferred to 4 L of XT medium for further cultivation at 70 °C for 5 days. Cells were harvested by centrifugation and washed with 0.85% NaCl solution. Washed cells were suspended in 10 mM Tris-HCl buffer (pH 8.0) containing 100 mM NaCl and 20 mM imidazole and disrupted by ultrasonication. Cell debris was removed by centrifugation (10,000× *g*, 10 min), and the supernatant was used as the crude extract. Enzyme purification was performed by using the AKTAprime Plus system (GE Healthcare). The crude extract was loaded on a HisTrap FF Crude column (5 mL, GE Healthcare) equilibrated with 10 mM Tris-HCl buffer (pH 8.0) containing 100 mM NaCl and 20 mM imidazole. The column was first washed with the same buffer and then washed again with three bed volumes of 10 mM Tris-HCl buffer (pH 8.0) supplemented with 20 mM imidazole. The enzyme was eluted by using a linear gradient of 20–500 mM imidazole in the same buffer over five bed volumes. Active fractions were pooled, and the solution was then loaded onto a HiTrap Q HP column (1 mL, GE Healthcare) equilibrated with 10 mM Tris-HCl buffer (pH 8.0). After the column was washed with the same buffer, the enzyme was eluted with a linear gradient of 0–500 mM NaCl in the same buffer. Active fractions were pooled and dialyzed against 10 mM Tris-HCl (pH 8.0), and the resultant enzyme solution was used for purified enzyme preparation.

### 4.7. Molecular Mass Determination of Protein

Molecular mass of the recombinant enzyme was determined by using native gradient PAGE on a Novex NativePAGE Bis-Tris gel system (Invitrogen, Carlsbad, CA, USA) according to the manufacturer’s instructions. Sodium dodecyl sulfate-polyacrylamide gel electrophoresis (SDS-PAGE) was performed on a 12.5% polyacrylamide gel containing 0.1% SDS according to the method previously described by Laemmli [28]. Samples were boiled for 5 min in 10 mM Tris-HCl buffer (pH 6.8) containing 1% SDS and 1% 2-mercaptoethanol. A WIDE-VIEW prestained protein size marker (Fujifilm Wako, Osaka, Japan) was used as the molecular mass standard. Protein bands were visualized by staining with Rapid CBB KANTO 3S (Kanto Kagaku, Tokyo, Japan) according to the manufacturer’s instructions.

### 4.8. Western Blot and N-Terminal Amino Acid Analysis

Western blotting analysis was performed by using a horseradish peroxidase-conjugated anti-His-tag antibody (Anti-His-tag mAb-HPR-DirecT, MBL, Nagoya, Japan). Samples were subjected to SDS-PAGE, followed by electroblotting to a polyvinylidene difluoride membrane (GE Healthcare). For signal detection, Blocking One (Nacalai Tesque, Kyoto, Japan) and TMB solution for Western blotting (Nacalai Tesque, Kyoto, Japan) were employed.

### 4.9. Determination of Prosthetic Group

Extraction and identification of the flavin compound from the enzyme were performed according to a previously described method [29]. PAGE was performed on a 5.5% polyacrylamide gel according to the method described by Davis [30]. Staining specific for non-heme iron and activity staining were performed as previously described [19,31].

### 4.10. Effect of Temperature and pH on Enzyme Activity and Stability

The optimal temperature for the enzyme reaction was determined by performing a standard assay at 40–80 °C. In order to determine the effect of temperature on enzyme stability, the enzyme was incubated for 10 min at different temperatures in 10 mM Tris-HCl buffer (pH 8.0). After centrifugation, residual activity in the supernatant was determined by using a standard assay method. In order to determine the effect of pH on enzyme stability, the enzyme was incubated in buffers of different pH for 30 min at 50 °C, and the remaining activity was determined by using the standard assay method. Buffers (100 mM) used for these assays were glycine-HCl (pH 2.0–3.0), sodium citrate-citrate (pH 3.0–4.0), acetate-sodium acetate (pH 4.0–5.5), MES-NaOH (pH 5.5–7.0), HEPES-NaOH (pH 7.0–8.0), Tris-HCl (pH 8.0–9.0), glycine-NaOH (pH 9.0–11.0), and Na_2_HPO_4_-NaOH (pH 11.0–12.0). In order to determine the optimal pH for enzyme activity, PIPES-NaOH (pH 6.0–7.0) and HEPES-NaOH (pH 6.5–8.0) buffers (200 mM) were used at 60 °C.

### 4.11. Kinetic Parameters

The Michaelis constant (Km) was determined from Michaelis–Menten plots by using the Solver tool in Microsoft Excel.

### 4.12. Preparation of a Dye-LDH Immobilized Electrode

A glassy carbon (GC) electrode (diameter: 3 mm) was continuously polished by using a diamond suspension (1.0 µm) and alumina suspension (0.05 µm alumina powder) and then rinsed with ultrapure water. After sonication in ultrapure water, the electrode was scanned 50 times at 100 mVs^−1^ across potentials ranging from −1.0 V to 1.0 V in 50 mM sulfuric acid. Finally, the electrode was washed with ultrapure water.

For electrode preparation, MWCNTs were dispersed in *N*-methyl-2-pyrrolidone (FUJIFILM Wako) as a 0.2% (*w*/*v*) solution. A 20 µL aliquot of MWCNT dispersion solution was cast on the GC electrode and dried at 50 °C for 3 h, and the process was repeated. In order to prepare a Dye-LDH immobilized electrode, a 20 µL aliquot of Dye-LDH (1 mg/mL) solution was dropped on the MWCNT modified electrode and dried at 25 °C for 1 h. For modification with PES (an electron mediator for Dye-LDH), the prepared Dye-LDH immobilized electrode was immersed in 450 µL of 0.4 mM arPES solution dissolved in 20 mM HEPES-NaOH (pH 7.5) and incubated at 25 °C for 3 h. After incubation, the electrode was washed twice with 20 mM HEPES-NaOH (pH 7.5) in order to remove unreacted arPES.

### 4.13. Electrochemical Measurements

Electrochemical measurements were performed by using an ALS electrochemical analyzer model 1205B (BAS Inc., Tokyo, Japan). A typical three-electrode system used in the study consisted of a Ag/AgCl (3 M KCl) electrode as the reference electrode, a platinum wire as the counter electrode, and a prepared GC electrode as the working electrode. The measurement temperature was set at 50 °C by using a temperature-controlled water bath. Cyclic voltammograms were recorded at a scan rate of 10 mV s^−1^ across a voltage range of −0.1 V to +0.5 V. The standard reaction mixture contained 200 mM PIPES-NaOH buffer (pH 6.5) and 40 mM l-lactate. l-Lactate was detected by using the CA method at a constant potential of 0 V vs. Ag/AgCl.

## Figures and Tables

**Table 1:** Purification of recombinant Dye-LDH from *S. acidocaldarius* SK1.

	Total Protein (mg)	Total Activity (units)	Specific Activity (units/mg)	Yield (%)	Fold Purification
Crude extract	477	6.99	0.0156	100	1.00
HisTrap FF Crude	1.51	0.684	0.453	9.79	28.9
HiTrap Q HP	0.441	0.676	1.53	9.67	98.1

**Table 2:** Primers used in this study.

Primer	Sequence
pET16540F	CCGCGCGGCAGCCATATGCTGGGAAAATTAATTTATGATA
pET16570R	TTAGCAGCCGGATCCTTAAGTAGTTTCGTATTTTATTTTT
pET15bF	ATGGCTGCCGCGCGGCACCAGGCCGCTGCT
pET15bR	GGATCCGGCTGCTAACAAAGCCCGAAAGGA
SSO16540F	GTGAAACCTTTAAGAATAGCATGGGCAGCAGCCATCATCA
SSO16570R	GGCCTCTTGCGGGATATCCGGATATAGTTC
pSSOF	AGCCATTGTCATAGTTTTCACTAGCTAAAA
pSSOR	GCTATTCTTAAAGGTTTCACATAAATAAAC
Fusion-pSSO	TGATGATGGCTGCTGCCCATGCTATTCTTAAAGGTTTCAC
pSAVF	AGCGCAACGCAATTAATGTGAGTTAGCTCA
pSAVR	CACTGCCCGCTTTCCAGTCGGGAAACCTGT
Infusion pSAV2F	GGAAAGCGGGCAGTGAGCCATTGTCATAGTTTTCA
Infusion pSAV2R	TAATTGCGTTGCGCTGGCCTCTTGCGGGATATCCG
16540FRT	GGGTAGAACAGTTGTCTAAGCTTAGTAAGA
16550RRT	AATATGTGAAGGTGGTTCATCAGCTAACTG
16550FRT	CTGTATTAGATGCGGAAGATGTCATCTTCA
16560FRT	GTAGTATATTAGCTCCGGTTATACCATTCC
16560RRT	TGAAAAGACTTTCACCATCTCCAATAGGAC
16570RRT	AGAAATCCATTAATTGTCACGTTCATGGGC

## Supplementary Materials

Supplementary materials can be found at https://www.mdpi.com/article/10.3390/ijms222413570/s1.

## Abbreviations

Dye-LDH	dye-linked l-lactate dehydrogenase
DCIP	2,6-dichlorophenolindophenol
FAD	flavin adenine dinucleotide
FMN	flavin mononucleotide
INT	p-iodonitrotetrazolium violet
MTT	3-[4,5-dimethylthiazol-2-yl]-2,5-diphenyltetrazolium bromide
MWCNT	multi-walled carbon nanotube
PES	phenazine ethosulfate
