# Supplementary material for: Characterization of a Novel Thermostable Dye-Linked l-Lactate Dehydrogenase Complex and Its Application in Electrochemical Detection
Source: Int J Mol Sci. 2021 Dec 17;22(24):13570. doi: 10.3390/ijms222413570 (PMC8704557; doi:10.3390/ijms222413570)
Supplement: Supplementary file 1 [file ijms-22-13570-s001.zip › ijms-1502792-supplementary.pdf]

International Journal of
Molecular Sciences

Supplementary Materials

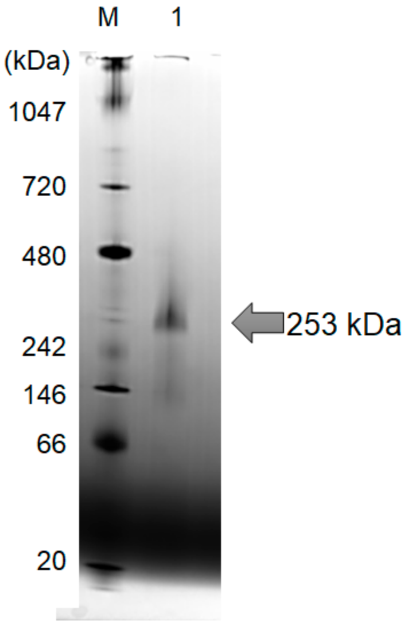

Supplementary Figure S1. Native gradient PAGE on a Novex NativePAGE Bis–Tris gel system of recombinant Dye-LDH. Lane M, markers; 1, the purified Dye-LDH.

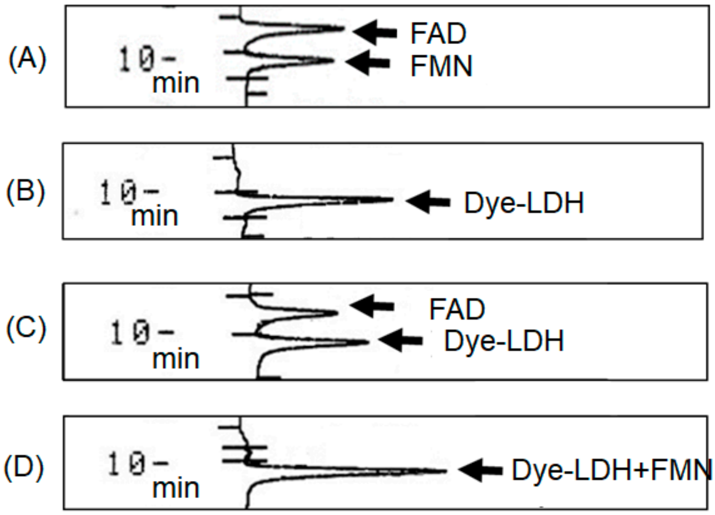

Supplementary Figure S2. HPLC analyses of the prosthetic groups. Elution profiles of the standard mixture (A), extract of Dye-LDH (B), FAD and extract of Dye-LDH (C), and FMN and extract of Dye-LDH (D).

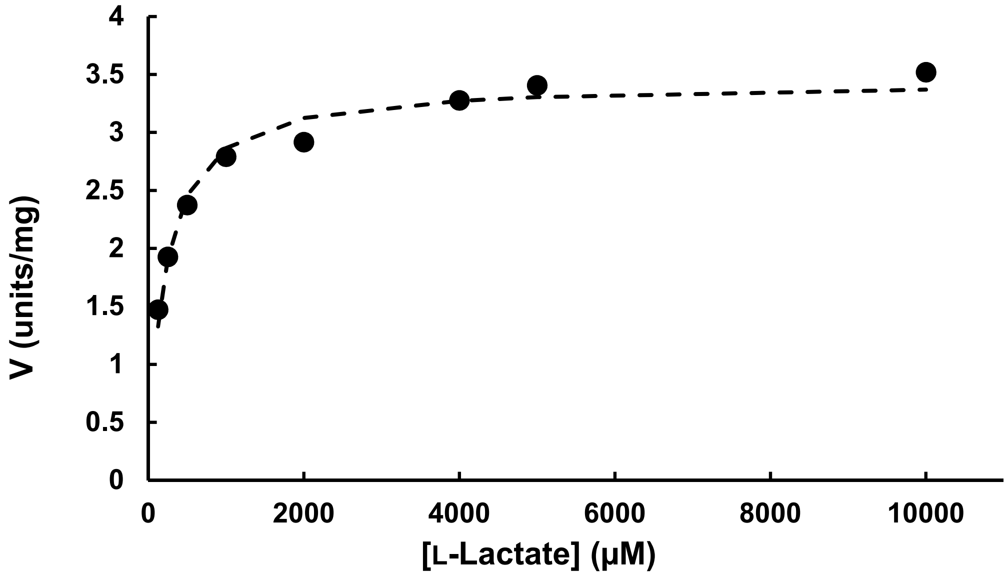

Supplementary Figure S3. Michaelis-Menten plot of Dye-LDH from Sf. tokodaii. Km was determined by the Solver tool of Microsoft Excel.

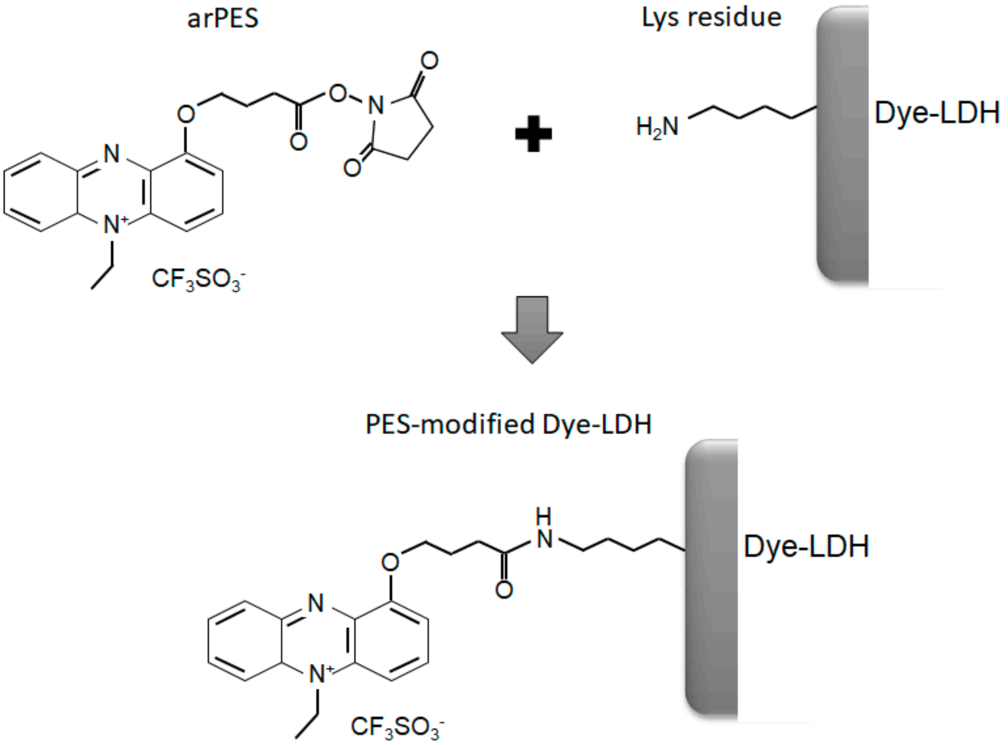

Supplementary Figure S4. The schematic diagram for the modification of Lys residue on Dye-LDH by succinimide group of arPES.
